# 

**Published:** 1872-10

**Authors:** 


### NOTICES.

HENRY WARD BEECHER'S "Yale Lectures on Preaching," published by J. B. Ford & Co., 27 Park Place, New York. These Lectures gain considerable interest from the fact that they come out as the first volume of a Uniform Author's Copyright Edition of Mr. Beecher's Works, to include "Lectures to Young Men," "Star Papers," "Summer in the Soul," "Royal Truths," "English and American Speeches," etc. These and others will be issued at short intervals, and form a set of books much called for by the public.

Also, by the same house, at $2.50 a volume, "The Sermons of Henry Ward Beecher," in Plymouth Church, Brooklyn, from verbatim reports, by T. J. Ellenwood, being the fifth and sixth series, from September, 1870, to September, 1871; the next thing to hearing the distinguished minister preach a whole year. No other Henry Ward Beecher lives. These discourses are full of practical suggestions as to the conduct of life, of inestimable value, especially to all high-minded young men and women, while Christians of every name will find answerings in their own hearts, which will help and encourage and strengthen them, in the faith once delivered to the saints.

We commend to the notice of our readers the "Aldine," $5.00 a year, the most beautifully illustrated monthly in this country. It shows a naturalness of conception, and a skill and delicacy of finish, without a parallel.

"The Model Potato," by JOHN McLAURIN, M. D., published by Samuel R. Wells, 389 Broadway, New York, and sent for twenty-five cents; referring to its proper cultivation, its disease and remedy, with directions for cooking it, making it worthy of a place in every kitchen in the world.

